# Effect of Supplementation with Probiotics in Patients with Schizophrenia: Systematic Review and Meta-Analysis of Randomized Controlled Clinical Trials

**DOI:** 10.3390/foods14101773

**Published:** 2025-05-16

**Authors:** Lu Li, Fengqi Du, Xilong Liu, Mengyao Song, Giuseppe Grosso, Maurizio Battino, Christine Boesch, He Li, Xinqi Liu

**Affiliations:** 1 Key Laboratory of Geriatric Nutrition and Health (Beijing Technology and Business University), Ministry of Education, School of Food and Health, Beijing Technology and Business University, Beijing 100048, China; 2230202097@st.btbu.edu.cn (F.D.); 2330202065@st.btbu.edu.cn (X.L.); sg999@st.btbu.edu.cn (M.S.); lihe@btbu.edu.cn (H.L.); 2 Department of Biomedical and Biotechnological Sciences, University of Catania, 95123 Catania, Italy; giuseppe.grosso@unict.it; 3 International Joint Research Laboratory of Intelligent Agriculture and Agri-Products Processing, Jiangsu University, Zhenjiang 212013, China; m.a.battino@staff.univpm.it; 4 Dipartimento di Scienze Cliniche Specialistiche e Odontostomatologiche, Università Politecnica delle Marche, Via Ranieri 65, 60130 Ancona, Italy; 5 Research Group on Food, Nutritional Biochemistry and Health, Universidad Europea del Atlántico, Isabel Torres 21, 39011 Santander, Spain; 6 School of Food Science and Nutrition, University of Leeds, Leeds LS2 9JT, UK; c.bosch@leeds.ac.uk

**Keywords:** probiotics, psychiatric symptom, lipid profile, glycemic indices, inflammation, oxidative stress, meta-analysis

## Abstract

Supplementation with probiotics seems to confer protective effects in individuals with schizophrenia (SZ), although available results are inconclusive. The aim of this study was to systematically review existing randomized clinical trials (RCTs) to critically assess the effect of probiotics on psychiatric symptoms, anthropometric indicators, lipid profiles, glycemic indices, inflammation, and oxidative stress in adults with SZ. A systematic search was conducted in four databases from inception until January 2025. Six RCTs were included in the quantitative analysis that demonstrated beneficial effects of probiotics on SZ severity determined via the Positive and Negative Syndrome Scale (PANSS), with significant reductions in PANSS (MD = −0.50, *p* = 0.001), PANSS Negative (MD = −0.31, *p* = 0.050), and PANSS General scores (MD = −0.33, *p* = 0.036), alongside reductions in body weight (MD = −0.92, *p* = 0.000), body mass index (MD = −0.53, *p* = 0.016), and total cholesterol (SMD = −0.34, *p* = 0.005). Furthermore, probiotic interventions reduced baseline glucose (SMD = −0.59, *p* = 0.000), insulin (MD = −0.68, *p* = 0.000), and measures of insulin sensitivity/resistance and significantly improved biomarkers of inflammation and oxidative stress. To summarize, this meta-analysis suggests that probiotics may confer beneficial effects in patients with SZ through improving psychiatric symptoms as well as markers of body weight, lipid and glucose metabolism, inflammation, and oxidative stress.

## 1. Introduction

Schizophrenia (SZ) is defined as a chronic and severe psychiatric disorder characterized by cognitive decline and physiological and emotional reduction [1]. SZ is a life-threatening disease, putting 1% of the world’s population in agony. The disease is closely related to genetic and environmental factors [2]. Accompanied by low short-term recovery rates, difficult prognoses, and poor employment rates, SZ is deeply concerning [3]. Antipsychotic medications have some ameliorative effects that can alleviate the disease symptoms, reduce relapse rates, and raise the standard of living [4]. However, the treatment of SZ with first- and second-generation antipsychotics has been reported to often be associated with side effects, including obesity and consequent increased risk of metabolic syndrome [5]. The main mechanisms underlying such trends are multifaceted, affecting several neurotransmitter systems that regulate hunger, satiety, and energy balance [6]. The affinity of various medicaments (e.g., olanzapine, quetiapine, and clozapine) to several central nervous system receptors, including serotonin, dopamine, histamine, and muscarinic receptors, is hypothesized to lead to sedation, increased appetite, higher susceptibility to cravings, and overeating [7]. Moreover, atypical antipsychotics (especially olanzapine and clozapine) may alter glucose and fat metabolism and hormonal dysfunction, with impairment of insulin sensitivity, increased leptin and ghrelin, and reduced adiponectin levels, resulting in increased fat storage and weight gain [8]. To date, there is no strong evidence of specific interventions to reduce the impact of overweight and cardiovascular disease. Hence, dietary counseling is highly warranted to guide patients toward healthy and conscious eating over the course of therapy.

The gut microbiome and the brain are strongly linked, influencing brain growth and development [9,10]. Consequently, the microbiota in the gut have an important impact on mood, stress, and the nervous system through gut–brain interactions [11]. Microbial metabolites such as short chain fatty acids (SCFAs) exert beneficial effects on psychiatric disorders via the microbiota–gut–brain axis through humoral, hormonal, and immune pathways [12]. Cytokines present in the gut are important immune system mediators, through the regulation of immune cells, exerting an impact on the central system as well as on the pathogenesis of SZ [13]. Furthermore, microbial metabolites stimulate the vagus nerve, thereby exerting an important impact on brain function in SZ [14]. The homeostasis of the gut microbiota is closely related to an individual’s mood, cognition, physiological condition, and mental state, and it has a moderating effect on psychiatric disorders through the interaction of the gut–brain axis. [15]. Probiotics have a beneficial action on the gut microbiota and play an extremely important role in restoring microbial composition, altering gut microbial diversity, boosting immunity, and ameliorating disease [16]. Animal studies suggest that probiotics can modulate endogenous neuroactive molecules [17], prevent and treat neuronal autoimmune diseases [18], and ameliorate impaired spatial memory in diabetic rats [19]. Notably, the gut microbiota may confer beneficial effects on SZ by regulating the expression of neurotrophic factor receptors and the levels of inflammatory factors and chemokines, thereby alleviating metabolic dysfunction and providing a new direction for obtaining beneficial effects in patients with SZ [20,21].

Although the relationship between the gut microbiome and SZ remains unclear [22], probiotics may result in some benefits in patients with SZ in terms of both somatization symptoms and collateral effects of canon therapeutic treatment [23]. This study was designed to explore the potential therapeutic role of probiotics in patients diagnosed with SZ through regulating psychiatric symptoms, anthropometric indicators, lipid profiles, glycemic indices, inflammation, and oxidative stress.

## 2. Experimental Section

### 2.1. Protocol and Registration

The present study was conducted in accordance with the Preferred Reporting Items for Systematic Reviews and Meta-Analyses (PRISMA) guidelines, as outlined in the following sections. It was registered under the number INPLASY202510113, and the associated DOI is 10.37766/inplasy2025.1.0113.

### 2.2. Search Strategy and Selection Criteria

A dual-reviewer approach was adopted for the literature search, screening, quality assessment, and data extraction, with L.L. serving as the arbiter in cases of conflicting opinions. The present process encompassed studies from inception up to January 2025 in the following bibliographic databases: the Cochrane Central Register of Controlled Trials (CENTRAL), PubMed, ScienceDirect, and Web of Science.

The literature search included the following terms: “probiotics” and names of individual strains, psychiatric symptom (“positive and negative syndrome scale (PANSS)” or “PANSS general” or “PANSS negative” or “PANSS positive” or “brief psychiatric rating scale (BPRS)”), anthropometric indicators (“body mass index (BMI)” and “weight”), lipid profile (“triglycerides (TG)” or “total cholesterol (TC)” or “low-density lipoprotein cholesterol (LDL-C)” or “very low-density lipoprotein cholesterol (VLDL-C)” or “high-density lipoprotein cholesterol (HDL)” or “total-/high-density lipoprotein cholesterol ratio”), glycemic indices (“fasting blood sugar” or “homeostasis model of assessed insulin resistance (HOMA-IR)” or “insulin” or “insulin resistance index (IRI)” or “quantitative insulin sensitivity check index (QUICK)”), inflammation (“high-sensitivity C-reactive protein (hs-CRP)”), oxidative stress (“total glutathione (GSH)” or “total antioxidant capacity (TAC)” or “malondialdehyde (MDA)”), “schizophrenia”. A bibliographic search was carried out by reviewing the reference lists of the included studies and the most important texts.

For inclusion, the following criteria applied: (1) randomized controlled trials, (2) assessing probiotic effects on changes in at least one outcome (psychiatric symptoms, anthropometric indicators, lipid profile, glycemic indices, inflammation, and oxidative stress) in adults with SZ, and (3) providing sufficient analyzable data. The exclusion criteria were as follows: (1) non-randomized controlled trials (2) with a duration of less than 6 weeks and (3) the intervention group including probiotics. This study reflected the effects of probiotics combined with other supplements, which may introduce confounding effects.

### 2.3. Study Selection and Quality Evaluation

#### 2.3.1. Data Collection Process

The information extracted for each study included the study name, country, population (number, age, and BMI), intervention group, control group, duration, and main outcome.

#### 2.3.2. Quality Evaluation

The Cochrane Collaboration’s Risk of Bias 2 (RoB2) tool was used to assess bias risk across five domains, rating each as “low risk”, “some concerns”, or “high risk” to evaluate methodological quality. Study quality was evaluated across five domains: randomization, intervention deviations, missing data, outcome measurement, and selective reporting [24]. As mentioned in Cochrane RoB 2.0, the differences between “some concerns” and “low risk” are as follows: the description of the randomization method was slightly vague or the blinding method was somewhat flawed but had little impact on the results of the trial or there was a high rate of missing data and correlation with the results was not analyzed or the subjective outcomes were not assessed using blinding but the criteria for assessing them were more clearly defined or a statement of the study protocol was missing, but the reported outcome indicators were reasonable and complete. Moreover, quality was assessed according to the following seven aspects using the Nutri Grade (Grading of Recommendations Assessment, Development, and Evaluation) scoring system: (1) risk of bias, study quality, and study limitations; (2) precision; (3) heterogeneity; (4) directness; (5) publication bias; (6) funding bias; (7) study design [25].

#### 2.3.3. Statistical Analysis

Statistical analysis was performed using Stata v14.0 and Review Manager 5.4 (The Cochrane Collaboration, London, UK, 2020) to calculate effect sizes via mean differences (MDs) or standardized mean differences (SMDs), using a random-effects model based on the DerSimonian–Laird method, yielding a more reliable overall effect estimate. Results are expressed as mean changes from baseline values. When data on changes were not explicitly provided in the RCTs, the SD was computed using the following formula: [SDpre^2^ + SDpost^2^ − 2 × Corr (pre, post) × SDpre × SDpost]^0.5^ (SDpre: SD before intervention; SDpost: SD after intervention; Corr (pre, post): within-participant correlation). An assumed correlation of 0.5 was used when unreported. SDs were calculated from standard errors or confidence intervals if unavailable. Study heterogeneity was evaluated via I^2^, categorized as low (I^2^ < 25%), moderate (25% ≤ I^2^ < 75%), or high (I^2^ ≥ 75%) [26].

To assess individual study influence, each was sequentially removed. Publication bias was evaluated using Egger’s test and funnel plot inspection, with statistical significance set at *p* < 0.05.

## 3. Results

### 3.1. Study Selection

**Figure 1:**
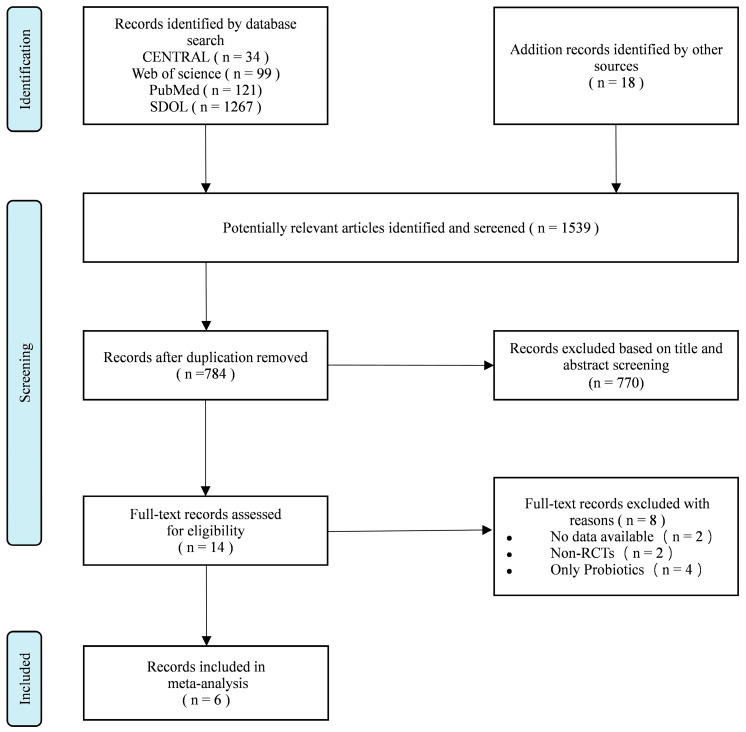
Flow diagram of the study selection, adapted from Ref. [27].

The process of study selection is illustrated in the PRISMA flow diagram presented in Figure 1. In total, 1539 publications were identified through searches conducted in databases and other relevant sources. After removing duplicates, 784 publications were retained. The titles and abstracts of the included studies were reviewed, resulting in the exclusion of 770 publications during this initial screening process. Following the full-text screening, six publications were included in the final meta-analysis.

### 3.2. Study Characteristics

Table 1 presents the characteristics of the six studies included in this analysis, encompassing a total of 361 patients diagnosed with SZ. Among these participants, 183 were assigned to the intervention group, while 178 were placed in the control group. All studies comprised both male and female participants. Participants in the intervention group received probiotics and selenium/vitamin D/dietary fiber/fructooligosaccharides.

### 3.3. Quality Evaluation

**Figure 2:**
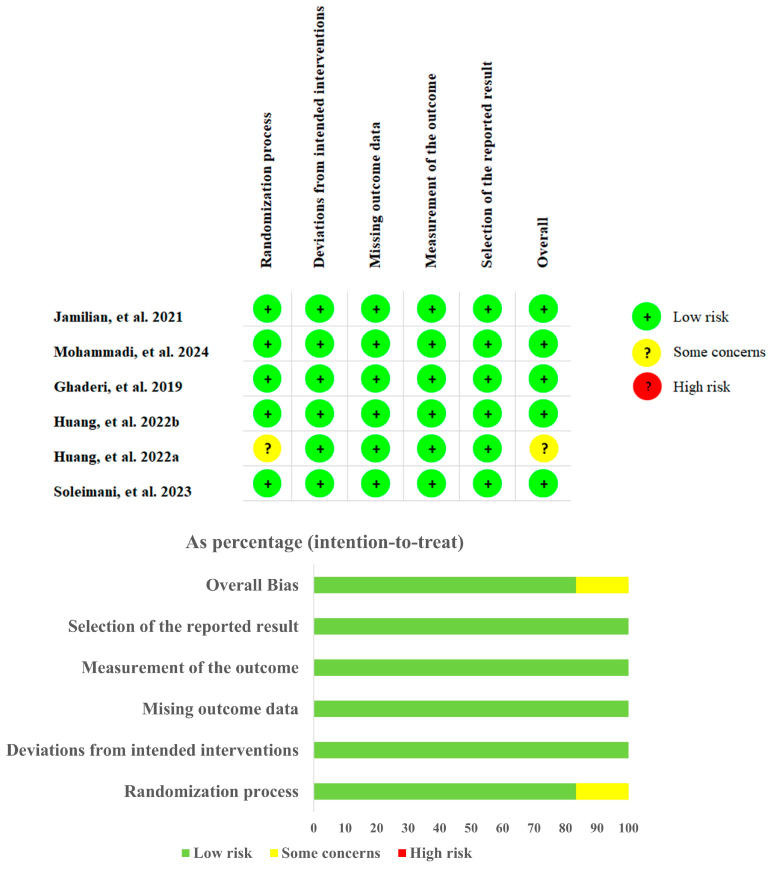
(**top**) Risk of bias summary. (**bottom**) Risk of bias graph [28,29,30,31,32,33].

A summary of the RoB2 tool and the risk of bias graphs is presented in (Figure 2). A total of six studies (100%) were classified as having no domains at “high risk”, while five studies (83.3%) demonstrated five domains of low risk. The quality of the present meta-analysis was evaluated based on the Nutri Grade scoring system, resulting in a score of 8.5, indicating high meta-evidence.

### 3.4. Outcomes

The effects of probiotic consumption on adults with SZ were evaluated in the present study. The results assessed included psychiatric symptoms (PANSS, PANSS Negative, PANSS General, PANSS Positive, and BPRS), anthropometric indicators (body mass index and weight), lipid profile (TGs, TC, LDL-C, VLDL-C, HDL-C, and total/HDL-cholesterol ratio), glycemic indices (glucose HOMA-IR, insulin IRI, and QUICKI), inflammatory biomarkers (hs-CRP), and oxidative stress (GSH, TAC, and MDA).

#### 3.4.1. Psychiatric Symptoms

**Figure 3:**
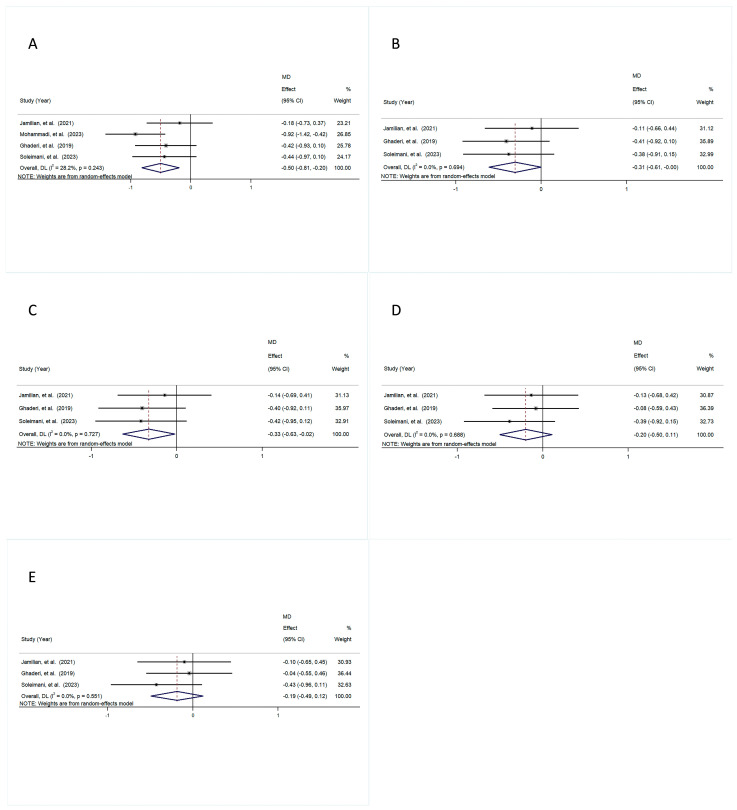
The forest plot is a visual representation of the effect of probiotic consumption: (**A**) PANSS; (**B**) PANSS Negative; (**C**) PANSS General; (**D**) PANSS Positive; (**E**) BPRS [28,29,30,33].

The pooled results revealed that the intervention group exhibited markedly diminished total PANSS scores in comparison with the control group (MD = −0.50, 95% CI [−0.81, −0.20], *p* = 0.001), with a small amount of heterogeneity (I^2^ = 28.2%, *p* = 0.243) (Figure 3A).

Similarly, the intervention group exhibited substantially lower PANSS Negative scores compared to the control group (MD = −0.31, 95% CI [−0.61, −0.00], *p* = 0.050), with a small amount of heterogeneity (I^2^ = 0.0%, *p* = 0.694) (Figure 3B).

Likewise, the results demonstrated a significant decrease in PANSS General scores in the intervention group compared to the control groups (MD = −0.33, 95% CI [−0.63, −0.02], *p* = 0.036), with low heterogeneity (I^2^ = 0.0%, *p* = 0.727) (Figure 3C).

In contrast, the results showed no statistically noticeable difference in PANSS Positive scores between the intervention and control groups, with an MD of −0.20 (95% CI [−0.50, 0.11], *p* = 0.204) and a small amount of heterogeneity (I^2^ = 0.0%, *p* = 0.688) (Figure 3D).

The comparison of the control and intervention groups revealed no significant difference in BPRS scores, with an MD of −0.19 (95% CI [−0.49, 0.12], *p* = 0.233) and a small amount of heterogeneity (I^2^ = 0.0%, *p* = 0.551) (Figure 3E).

#### 3.4.2. Anthropometric Indicators

**Figure 4:**
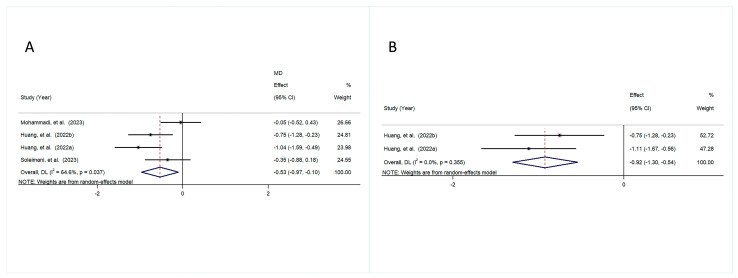
The forest plot is a visual representation of the effect of probiotic consumption: (**A**) BMI; (**B**) weight [31,32,33].

The pooled results demonstrated that there was a significant decrease in BMI in the intervention group compared with the control group (MD = −0.53, 95% CI [−0.97, −0.10], *p* = 0.016), with a moderate amount of heterogeneity (I^2^ = 64.6%, *p* = 0.037) (Figure 4A).

A significant decline in weight in the intervention group was observed compared with the control group (MD = −0.92, 95% CI [−1.30, −0.54], *p* = 0.000), with a small amount of heterogeneity (I^2^ = 0.0%, *p* = 0.355) (Figure 4B). 

#### 3.4.3. Lipid Profile

**Figure 5:**
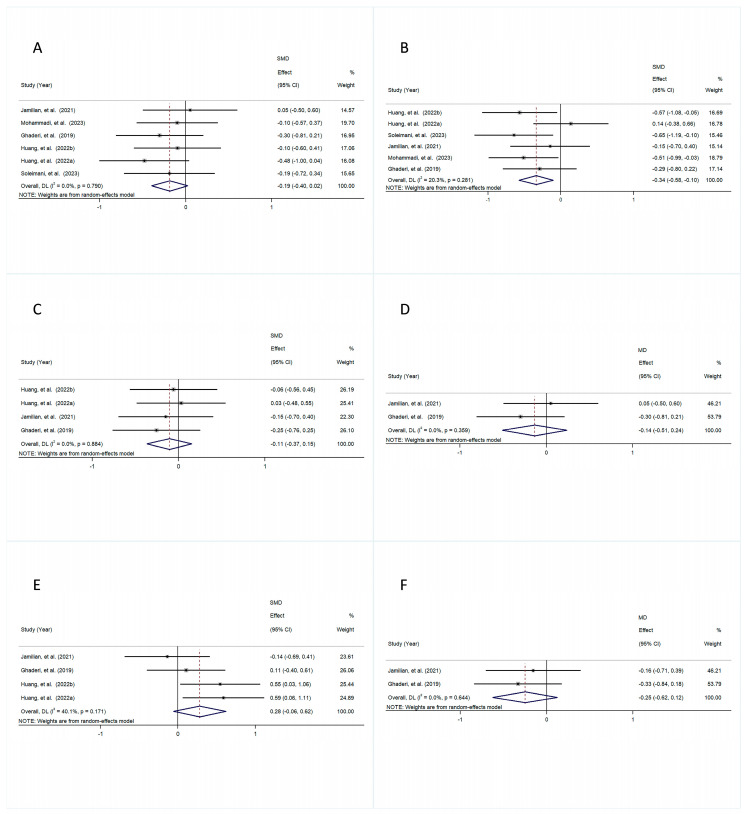
The forest plot is a visual representation of the effect of probiotic consumption: (**A**) TGs; (**B**) TC; (**C**) LDL-C; (**D**) VLDL-C; (**E**) HDL-C; (**F**) total/HDL-cholesterol ratio [28,29,30,31,32,33].

The pooled results demonstrated that a declining trend in TG levels was observed in the intervention group compared to the control group, with an SMD of −0.19 (95% CI [−0.40, 0.02], *p* = 0.082) and a small amount of heterogeneity (I^2^ = 0.0%, *p* = 0.790) (Figure 5A).

Meanwhile, there was a significant reduction in TC levels revealed in the intervention group in comparison with the control group (SMD = −0.34, 95% CI [−0.58, 0.10], *p* = 0.005), with a small amount of heterogeneity (I^2^ = 20.3%, *p* = 0.281) (Figure 5B).

A comparative analysis of the intervention group and the control group showed no substantial difference in LDL-C (SMD = −0.11, 95% CI [−0.37, 0.15], *p* = 0.423), with a small amount of heterogeneity (I^2^ = 0.0%, *p* = 0.884) (Figure 5C).

The results showed no noticeable difference in VLDL-C in the intervention group compared to the control group, with an MD of −0.14 (95% CI [−0.51, 0.24], *p* = 0.475) and a small amount of heterogeneity (I^2^ = 0.0%, *p* = 0.359) (Figure 5D).

Likewise, the results demonstrated no statistically significant difference in HDL-C between the intervention and control groups, with an SMD of 0.28 (95% CI [−0.06, 0.62], *p* = 0.105) and a moderate amount of heterogeneity (I^2^ = 40.1%, *p* = 0.171) (Figure 5E).

No changes were observed in the total/HDL-cholesterol ratio between the intervention and control groups (MD = −0.25, 95% CI [−0.62, 0.12], *p* = 0.189), with a small amount of heterogeneity (I^2^ = 0.0%, *p* = 0.644) (Figure 5F).

#### 3.4.4. Glycemic Indices

**Figure 6:**
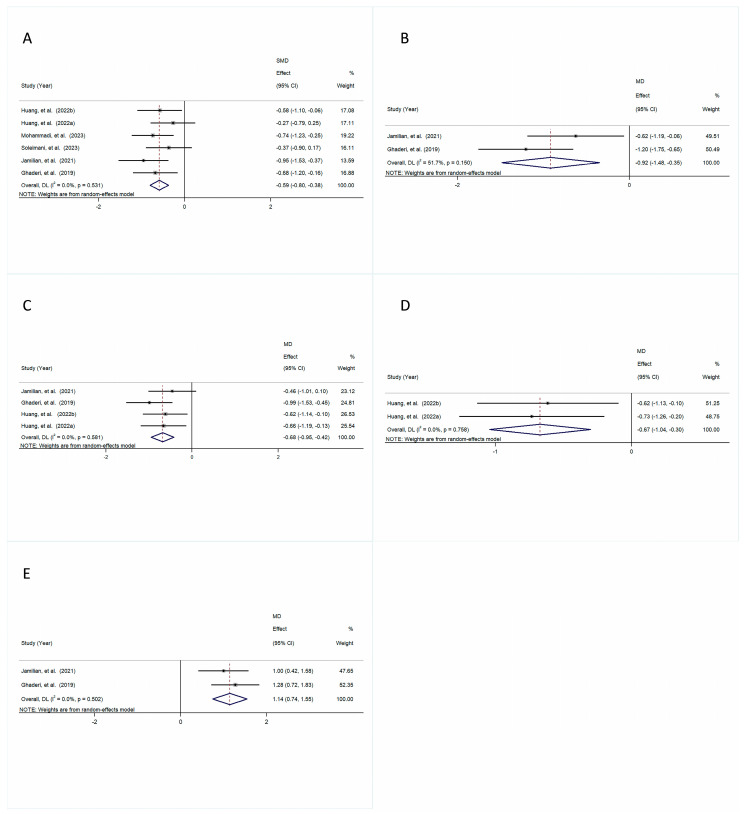
The forest plot is a visual representation of the effect of probiotic consumption: (**A**) glucose; (**B**) HOMA-IR; (**C**) insulin; (**D**) IRI; (**E**) QUICKI [28,29,30,31,32,33].

A pooled analysis was conducted, which revealed a significant decrease in fasting glucose levels in the intervention group when compared to the control group. The mean difference (SMD) was −0.59 (95% confidence interval [−0.80, −0.38], *p* = 0.000), and the heterogeneity was found to be low (I^2^ = 0.0%, *p* = 0.531) (Figure 6A).

Furthermore, a substantial decrease in HOMA-IR was observed in the intervention group in comparison with the control group, exhibiting an MD of −0.92 (95% CI [−1.48, 0.35], *p* = 0.002). The study also revealed moderate heterogeneity (I^2^ = 0.517, *p* = 0.150) (Figure 6B).

The findings demonstrated a significant decline in insulin levels within the intervention group, in contrast to the control group. The MD was found to be −0.68 (95% CI [−0.95, −0.42], *p* = 0.000), with low heterogeneity (I^2^ = 0.0%, *p* = 0.581) (Figure 6C).

The group that received the intervention also demonstrated a significant reduction in IRI in comparison with the control group (MD: = −0.67, 95% CI [−1.04, −0.30], *p* = 0.000), with minimal heterogeneity (I^2^ = 0.0%, *p* = 0.758) (Figure 6D).

A comparative analysis of the intervention and control groups revealed that probiotic supplementation significantly enhanced QUICKI (MD = 1.14, 95% CI [0.74, 1.55], *p* = 0.000), with minimal heterogeneity (I^2^ = 0.0%, *p* = 0.502) (Figure 6E).

#### 3.4.5. Inflammation

**Figure 7:**
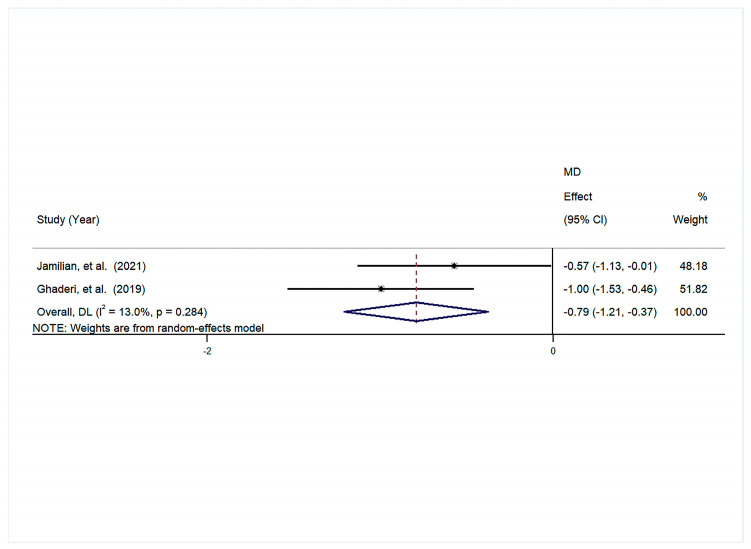
The forest plot is a visual representation of the effect of probiotic consumption on inflammation [28,30].

The pooled results showed that a significant reduction was observed in hs-CRP in the intervention group compared to the control group (MD = −0.79, 95% CI [−1.21, −0.37], *p* = 0.000), with a small amount of heterogeneity (I^2^ = 13.0%, *p* = 0.284) (Figure 7).

#### 3.4.6. Oxidative Stress

**Figure 8:**
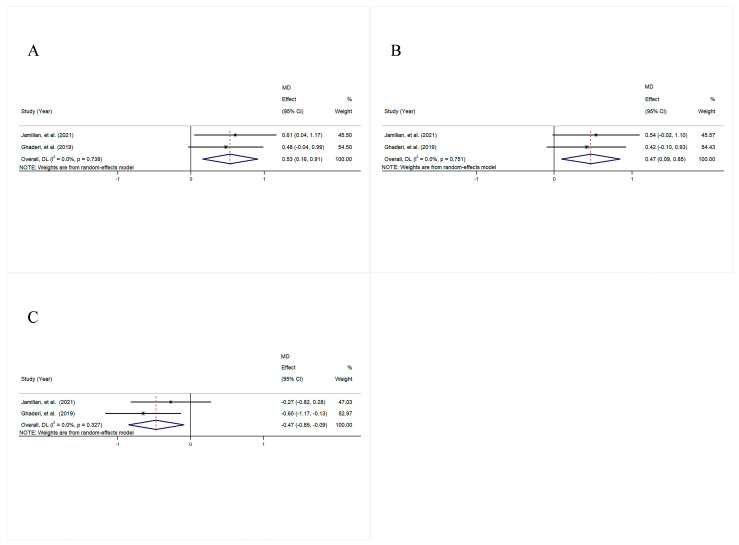
The forest plot is a visual representation of the effect of probiotic consumption: (**A**) GSH; (**B**) TAC; (**C**) MDA [28,30].

The pooled analysis showed that the plasma GSH in the intervention group significantly increased compared with the control group (MD = 0.53, 95% CI [0.16, 0.91], *p* = 0.006), with a small amount of heterogeneity (I^2^ = 0.0%, *p* = 0.738) (Figure 8A).

Similarly, the intervention group exhibited a marked increase in TAC levels in comparison with the control group, with an MD of 0.47 (95% CI [0.09, 0.85], *p* = 0.014) and a small amount of heterogeneity (I^2^ = 0.0%, *p* = 0.751) (Figure 8B).

In contrast, a significant decline in MDA was observed in the intervention group compared to the control group (MD = −0.47, 95% CI [−0.85, −0.09], *p* = 0.014), with a small amount of heterogeneity (I^2^ = 0.0%, *p* = 0.327) (Figure 8C). Sensitivity analysis of markers of psychiatric symptoms, anthropometric indicators, lipid profiles, glycemic indices, inflammation, and oxidative stress demonstrated that the overall effect did not change, and the funnel plots indicated that there was no publication bias (Figures S1–S6) 

## 4. Discussion

The PANSS scale comprises three distinct scales: a positive scale, a negative scale, and a general scale. The minimum clinically important difference (MCID) was used as the value of change in clinical efficacy, with a reduction in PANSS ≥ the MCID threshold indicating effectiveness. MCIDs ranged from 14.02 to 31.50 for PANSS [34]. This study revealed that consumption of probiotics in the context of SZ therapy markedly reduced total PANSS, PANSS General, and PANSS Negative. Higher scores are associated with more severe symptoms of SZ [35]. A prior investigation demonstrated that when patients with SZ consumed Bifidobacterium breve A-1 for a period of 4 weeks, there was a significant reduction in PANSS General subscale scores [36]. However, the use of *Lactobacillus rhamnosus* strain *GG* and *Bifidobacterium animalis* subsp. *lactis* strain *Bb12* did not have an effect on PANSS scores in SZ patients in a 14-week trial [37]. In contrast to previous studies, the current study showed that probiotics mitigated the severity in psychiatric symptoms of SZ. However, probiotics were less effective compared to standard medications.

Patients with SZ tend to experience cardiometabolic comorbidities due to higher rates of obesity caused by a poor lifestyle and the adverse effects of medication [38]. This meta-analysis demonstrates that probiotics seem to be effective in the reduction of body weight and BMI. Similarly, 12-week supplementation with Bifidobacterium breve B-3 (5 × 10^10^ CFU) resulted in considerable decrease in fat mass in obese adults [39]. Probiotic supplementation significantly reduced body weight and BMI, waist circumference, fat mass, and fat percentage, according to a meta-analysis [40]. In line with these findings, a 12-week intervention using *HY7601* and *KY1032* demonstrated a significant decrease in weight, body fat, and L1 subcutaneous fat area in overweight individuals [41]. Likewise, *HY7601* and *KY1032* also demonstrated the ability to mitigate weight gain and fat accumulation in mice that were rendered obese through a high-fat diet regimen [42].

Notably, SZ is associated with increased risk of dyslipidemia [43]. This study showed that probiotics considerably decreased TC in patients with SZ, albeit not total/HDL-cholesterol ratio, HDL-C, LDL-C, TGs, or VLDL-C. In support of these findings, 8 weeks of exposure to a formulation of *L. ferment* inhibited lipid accumulation and reduced TC and increased HDL-C in a maternal dyslipidemia rat model [44]. Similarly, a prior study highlighted that multispecies probiotics resulted in a notable reduction in TGs and total TC among patients with type 2 diabetes [45]. However, this intervention did not have a significant impact on LDL-C or HDL-C levels. Specifically, subgroup analyses highlighted the more evident role played by powdered and multistrain probiotics in regulating TC and TGs. Single *Lactobacillus*, *Bifidobacterium*, or *Pediococcus* had a regulatory effect on dyslipidemic populations, lowering BMI, TC, and LDL and increasing HDL [46]. In contrast, *Lactobacillus* demonstrated a significant regulatory effect in lowering TGs.

There is a robust correlation between the disorders of glucose metabolism and SZ [47]. This study showed that probiotic consumption led to a decrease in insulin, IRI, HOMA-IR, and glucose, while markedly increasing QUICKI in patients with SZ. Similarly, multiple species of probiotics significantly alleviated hyperglycemia in participants with type 2 diabetes mellitus, with significant effects on fasting blood sugar and HOMA-IR [48]. Specifically, subgroup analyses showed more significant modulations of FBS and HMOA-IR in patients aged <55 years with a BMI < 30 kg/m^2^ and an intervention period >8 weeks. Furthermore, composite strains exerted a pronounced influence on glucose metabolism in pregnant women when compared to single strains [49]. In support of these findings, 12-week supplementation with vitamin D3 and probiotics, including *Lactobacillus acidophilus*, *Bifidobacterium bifidum*, *Lactobacillus reuteri*, and *Lactobacillus fermentum*, resulted in beneficial effects on plasma NO, TAC, glycemia, and HDL-C in type 2 diabetic individuals with coronary heart disease [50]. Multistrain synergy of probiotic complexes plays a pivotal role in glycemic control.

The important role of inflammation in SZ has received much attention. Increased stress and lowered immunity promote the expression of pro-inflammatory factors, which ultimately lead to the development of chronic inflammation. Low-level inflammation exacerbates the symptoms of SZ through dopaminergic, serotonergic, noradrenergic, and glutamatergic neurotransmission [51]. Recent studies have demonstrated a negative correlation between cognitive function in individuals diagnosed with SZ and the levels of inflammatory markers, including IL-6, IL-1β, TNF-α, and CRP [52]. This finding underscores the importance of investigating the regulatory mechanisms of inflammation as a potential therapeutic approach for SZ and related conditions. The current meta-analysis showed that hs-CRP was significantly decreased in patients with SZ after probiotic supplementation, likely due to the anti-inflammatory effects of probiotics. Similarly, supplementation with *Lactobacillus rhamnosus GG* (2 × 10^9^ CFU day^−1^) for 4 weeks inhibited TNF-α, IL-6, IL-1β, and IL-17A levels and increased IL-10 and TGF-β levels in mice exposed to PM2.5 [53]. Furthermore, probiotics significantly decreased IL-4, IL-6, IL-12, hs-CRP, and TNF-α in adults, according to a meta-analysis [54]. This suggests that probiotics have a strong modulating effect on inflammation.

Impaired antioxidant capacity is observed in schizophrenic patients. Antipsychotic drugs can relieve this symptom; however, they contribute to some side effects [55]. Probiotics are food-derived antioxidants with strong antioxidant properties as well as strain specificity [56]. The present meta-analysis demonstrated that probiotics significantly increased GSH and TAC and markedly decreased MDA. In support of these findings, a meta-analysis showed that multiprobiotic supplementation counteracted oxidative damage in D-galactose-induced mice, increased plasma SOD as well as serum GSH-PX levels, and decreased serum MDA [57]. A mixture of *Lactobacillus* and *Bifidobacterium* preparations was shown to attenuate oxidative damage in mice with Alzheimer’s disease by elevating SIRT1, reducing the amount of RARβ-acetylated lysine and levels of p53 [58]. Similarly, a recent meta-analysis showed that probiotics or synbiotics increased TAC and GSH, while they reduced MDA levels in adults [59]. Specifically, subgroup analysis indicated that TAC and NO significantly increased in adults ≤50 years of age and that NO, GSH, and TAC were significantly elevated in overweight adults.

The funnel plots of the results demonstrated a symmetrical distribution, indicating that there was no significant publication bias. Likewise, the scatter points in the Egger regression plot were symmetrically distributed on both sides of the regression line, and the overall trend remained symmetrical, indicating that there was no significant publication bias. The sensitivity analyses showed small fluctuations in the effect size estimates after item-by-item exclusion, indicating that the results were robust.

Limitations of this study need to be acknowledged. Firstly, the number of original studies included in the analysis was limited (only six RCTs), and the study sample sizes were small. Secondly, co-supplementation (e.g., selenium, vitamin D, dietary fiber, or oligofructose) may have contributed to the observed effects, making it difficult to differentiate whether the beneficial effects stemmed from probiotics alone or the synergistic effects of multiple supplement types. Thirdly, differences in baseline parameters, the use of different probiotics, and differences in dosages may have contributed to the heterogeneity between studies. Furthermore, there was a lack of long-term follow-up data, given that the duration of the studies was 12 weeks. Long-term effects still warrant investigation.

## 5. Conclusions

This meta-analysis indicates that probiotics combined with other supplements may exert favorable effects on patients with SZ through improvements in psychological condition (PANSS, PANSS Negative, and PANSS General scores), anthropometric markers (BMI and weight), the harmonization of lipid metabolism (TC), the regulation of glycemic indices (glucose, HOMA-IR, insulin, IRI, and QUICKI), the attenuation of inflammation (hs-CRP), and the alleviation of oxidative stress (GSH, TAC, and MDA). Despite the complexity of the underlying mechanisms contributing to SZ, this meta-analysis demonstrates that probiotics seem to exert beneficial effects on patients with SZ. It also provides extensive information for future well-designed RCTs.

## Figures and Tables

**Table 1:** Features of the studies included in the meta-analysis.

Study	Country	Population			Intervention Group	Control Group	Duration (Weeks)	Main Results
		N (IG/CG)	Age	BMI				
[28]	Iran	25/26	18–60	IG: 25.1 ± 4.4 CG: 24.5 ± 3.0	200 μg/day selenium plus 8 × 10^9^ CFU/day of probiotic supplements (equal amounts of *L. acidophilus*, *B. lactis*, *B. bifidum*, and *B. longum*).	Placebo	12	↑: TAC, GSH, QUICKI ↓: hs-CRP, fasting glucose, insulin, HOMA-IR
[29]	Iran	35/35	18–65	IG: 27.26 ± 5.03 CG: 25.50 ± 6.37	2 × 10^9^ CFU/day of *Lactobacillus acidophilus*, *Lactobacillus rhamnosus*, *Lactobacillus reuteri*, *Lactobacillus paracasei*, *Bifidobacterium longum*, and *Bacillus coagulans* and 400 IU vitamin D.	Placebo	12	↑: MOCA ↓: TC, FBS, CRP
[30]	Iran	30/30	25–65	IG: 23.1 ± 2.8 CG: 24.5 ± 3.7	50,000 IU of vitamin D3 every 2 weeks plus 8 × 10^9^ CFU/day of probiotics containing *Lactobacillus acidophilus*, *Bifidobacterium bifidum*, *Lactobacillus reuteri*, and *Lactobacillus fermentum* (each 2 × 10^9^ CFU/day).	Placebo	12	↑: TAC, PANSS General, Total PANSS ↓: MDA, hs-CRP, FPG, insulin, HOMA-IR, TGs, TC
[31]	China	32/28	18–45	IG: 5.02–27.48 CG: 26.18–29.05	1.7 × 10^9^ CFU/g of *Bifidobacterium*, 3.8 × 10^8^ CFU/g of *Lactobacillus*, and 7.8 × 10^8^ CFU/g of *Enterococcus*, in total (1680 mg/d), plus dietary fiber (60 g/d).	Placebo	12	↓: Weight, BMI, TC
[32]	China	30/28	18–50	IG: 20.04 ± 2.84 CG: 21.12 ± 1.56	*Bifidobacterium*, *Lactobacillus*, and *Enterococcus* at concentrations ≥5.0 × 10^7^ CFU/g, in total (840 mg twice daily), plus dietary fiber (30 g twice daily).	Placebo	12	↓: Weight, IRI
[33]	Iran	31/31	18–60	IG: 27.3 ± 2.6 CG: 27.5 ± 2.9	38.5 mg fructooligosaccharides, 9 × 10^9^ CFU/g of *Lactobacilli*, 1.25 × 10^10^ CFU/g of *Bifidobacteria* plus 1.5 × 10^10^ CFU/g of *Streptococcus Salivarius* subsp. *Thermophilus*, in total (500 mg daily).	Placebo	12	↓: TGs, TC, FBS

TAC, total antioxidant capacity; GSH, total glutathione; QUICKI, quantitative insulin sensitivity check index; hs-CRP, high-sensitivity C-reactive protein levels; HOMA-IR, homeostasis model of assessment—insulin resistance; MOCA, Montreal Cognitive Assessment; TC, total cholesterol; TGs, triglycerides; FBS, fasting blood sugar; CRP, C-reactive protein; MDA, malondialdehyde; FPG, fasting plasma glucose; HOMA-IR, homeostasis model of assessment—estimated insulin resistance; PANSS, Positive and Negative Syndrome Scale; BMI, body mass index; IRI, insulin resistance index.

## Data Availability

The original contributions presented in the study are included in the article/Supplementary Materials, further inquiries can be directed to the corresponding authors.

## Supplementary Materials

The following supporting information can be downloaded at: https://www.mdpi.com/article/10.3390/foods14101773/s1, Figures S1–S6.
