# Supplementary figures and images for: Effect of Supplementation with Probiotics in Patients with Schizophrenia: Systematic Review and Meta-Analysis of Randomized Controlled Clinical Trials
Source: Foods. 2025 May 16;14(10):1773. doi: 10.3390/foods14101773 (PMC12111037; doi:10.3390/foods14101773)

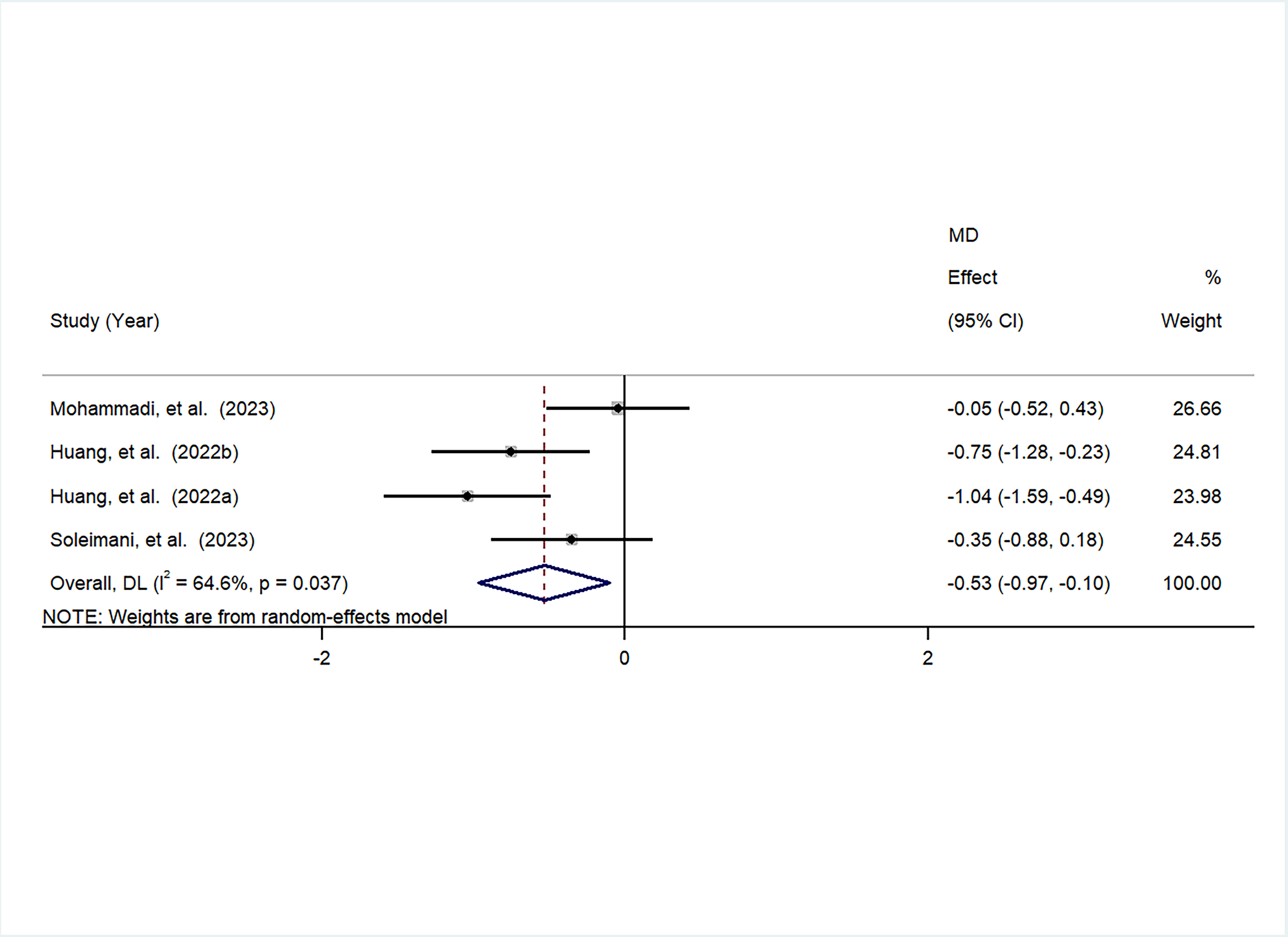

Supplement: Supplementary file 1 [file foods-14-01773-s001.zip › Appendix A1-A6 pictures/Fig A2 1.1.tif]
